# The First Evaluation of Serum Levels of MGP, Gas6 and EGFR after First Dose of Chemotherapy in Lung Cancer

**DOI:** 10.3390/biology11010082

**Published:** 2022-01-06

**Authors:** Andreea Crintea, Alina Gabriela Dutu, Anne-Marie Constantin, Zsolt Fekete, Gabriel Samasca, Iulia Lupan, Ioan Alexandru Florian, Ciprian Nicolae Silaghi, Alexandra Marioara Craciun

**Affiliations:** 1 Department of Medical Biochemistry, Iuliu Hațieganu University of Medicine and Pharmacy, 400349 Cluj-Napoca, Romania; crintea.andreea@umfcluj.ro (A.C.); Alina.Dutu@umfcluj.ro (A.G.D.); silaghi.ciprian@umfcluj.ro (C.N.S.); acraciun@umfcluj.ro (A.M.C.); 2 Department of Morphological Sciences, Iuliu Hațieganu University of Medicine and Pharmacy, 400349 Cluj-Napoca, Romania; 3 Zsolt Fekete, Department of Oncology, Iuliu Hațieganu University of Medicine and Pharmacy, 400349 Cluj-Napoca, Romania; fekete.zsolt@umfcluj.ro; 4 Department of Immunology, Iuliu Hațieganu University of Medicine and Pharmacy, 400349 Cluj-Napoca, Romania; 5 Interdisciplinary Institute of BioNanoScience, 400006 Cluj-Napoca, Romania; iulia.lupan@ubb.cluj.ro; 6 Department of Neurosurgery, Iuliu Hațieganu University of Medicine and Pharmacy, 400349 Cluj-Napoca, Romania; florian.ioan@umfcluj.ro

**Keywords:** MGP, Gas6, EGFR, Vitamin K, chemotherapy, lung cancer

## Abstract

**Simple Summary:**

Serum levels of MGP, Gas6, vitamin K_1_, and EGFR were not significantly changed in response to the first cycle of chemotherapy. We found a strong correlation between MGP and VitK_1_ serum values, and a moderate negative correlation between VitK_1_ and EGFR in pre-treatment patients. The post-treatment value of EGFR is a strong independent factor that correlates positively with the Gas6 post-treatment values.

**Abstract:**

Background: Vitamin K-dependent proteins (VKDPs) and the epidermal growth factor receptor (EGFR) are involved in lung cancer progression. Therefore, we aimed to study the serum concentration of Matrix Gla protein (MGP), Growth Arrest-specific 6 (Gas6), and EGFR before and after the first cycle of chemotherapy and to investigate how MGP, Gas6, and EGFR are modified after one cycle of chemotherapy. Methods: We performed an observational study on twenty patients diagnosed with lung cancer, by assessing the serum concentration of vitaminK_1_ (VitK_1_), MGP, Gas6, and EGFR using the ELISA technique before and after three weeks of the first cycle of chemotherapy. Patients were evaluated using RECIST 1.1 criteria. Results: Serum levels of MGP, Gas6, EGFR, and VK_1_ before and after treatment were not changed significantly. Regarding the pre-treatment correlation of the MGP values, we found a strong positive relationship between MGP and VK_1_ pre-treatment values (r = 0.821, 95%CI 0.523; 0.954, *p* < 0.001). Furthermore, there was a moderately negative correlation between VK_1_ and EGFR pre-treatment values, with the relationship between them being marginally significant (r = −0.430, 95%CI −0.772; 0.001, *p* = 0.058). Post-treatment, we found a strong positive relationship between MGP and VK_1_ post-treatment values (r = 0.758, 95%CI 0.436; 0.900, *p* < 0.001). We also found a moderate positive relationship between Gas6 and EGFR post-treatment values, but the correlation was only marginally significant (r = 0.442, *p* = 0.051).

## 1. Introduction

The deaths caused by lung cancer represented about 18% of the total number of deaths worldwide in 2020, for both sexes and all ages. Lung cancers are divided into two main groups: small-cell carcinoma (SCLC, representing 13% of all cases) and non-small-cell carcinoma (NSCLC, representing 83% of all cases) [1,2,3,4,5]. Molecular profiling further classifies NSCLC into adenocarcinoma (ADC) and its variants, squamous cell carcinoma (SqCC), large-cell lung carcinoma (LCLC), and other types (salivary gland-type tumors, sarcomatous carcinomas, and not otherwise specified) [6]. Originating in the bronchial mucous glands, ADC is the most common type of NSCLC, representing the most common subtype of lung cancer in non-smokers [7].

Located in various tissues such as bones, blood vessels, or the heart, 17 vitamin K-dependent proteins have been identified until now [8]. The VKDPs are well known for their involvement in coagulation by factors II, VII, IX, and X. The VKDPs become active through the carboxylation process, which occurs in the liver (for the coagulation factors mentioned earlier) or peripherally, within the blood vessels [5]. The gamma-glutamyl carboxylase enzyme is needed for the full functionality of VKDPs and acts in a vitamin K-dependent manner, both hepatically and extrahepatically [6].

In recent years, the involvement of the VKDPs in lung cancer has become intensely studied, with recent studies reporting especially the role in tumor progression of Gas6 and MGP [7,8]. Furthermore, the role of EGFR in lung cancer is better established as it is already a targeted therapy [9].

MGP is a 10-kDa calcium-binding matrix protein that is secreted and contains several post-translationally modified-carboxyl-glutamic acid residues resulting from vitamin K-dependent carboxylation [4] In vitro, MGP links its Gla residues to calcium and hydroxyapatite to function as a critical regulator/inhibitor of mineralization [10].

High circulating levels of MGP in tumors have been linked to tumor aggressiveness and poor prognosis in breast cancer, glioblastoma [11], primary renal-cell carcinomas, prostate carcinomas, and testicular germ-cell tumors [12]. Furthermore, it was observed that osteosarcoma patients that developed lung metastases had high serum levels of MGP at diagnosis. The MGP action of favoring metastasis spread can be explained by its effects of altering endothelial adhesion and the tumor cell’s extravasation ability and by modulating metalloproteinase activities [13]. In NSCLC, it was observed that MGP gene expression could promote macrophage recruitment, neovascularization, and tumor growth [14].

Gas6 has a molecular weight of 75 kDa and a length of 721 amino acids [15]. Gas6 has many physiological roles, of which tissue repair, inflammation, cell survival, proliferation, and migration are worth mentioning [16]. In terms of its role in lung diseases, Gas6/AXL signaling has been shown to reduce alveolar inflammation and acute ischemia-reperfusion injury [17].

In lung ADC, the Gas6-stimulated phosphorylation of the AXL [a tyrosine kinase receptor (RTK) with oncogenic potential and transforming activity] is enhanced [18]. Gas6, derived from CAFs (carcinoma-associated fibroblasts), may promote the migration of AXL-expressing lung cancer cells during chemotherapy. Given that patients with tumor AXL- and stromal Gas6-positive tumors had significantly lower five-year disease-free survival rates than the double negative group, it was concluded that Gas6 is involved in poor clinical outcomes [19]. Therefore, AXL/Gas6 might represent prognostic biomarkers for identifying cases at high risk of post-operative death [20]. Ultimately, it was observed that high AXL mRNA expression [21] and low expression of long noncoding RNA Gas6-AS1 [22] may predict poor prognosis in patients with NSCLC.

EGFR, also known as ERBB1 [Epidermal growth factor (EGF) receptor 1] and HER1 (Human EGFR related 1), is an RTK and a crucial component of several cell signaling pathways. Its binding to ligands (EGF and TGF-) activates three major downstream signaling pathways (RAS/RAF/MAPK, PI3K/AKT, and JAK/STAT), stimulating mitosis and inhibiting apoptosis. The RAS/RAF/MAPK, PI3K/AKT, and JAK/STAT pathways play important roles in normal cell growth, proliferation, survival, and differentiation [23].

EGFR gene mutations and protein overexpression are associated with lung cancer, especially with NSCLC [23]. EGFR and KRAS (Kristen rat sarcoma virus) mutations are frequently found in lung ADCs [24]. Secondary mutations in EGFR (such as T790M) or upregulation of the MET kinase are found in over 50% of resistant tumors [25]. The importance of EGFR in lung cancers supports the concept of “oncogene addiction” [26]. Patients with EGFR mutations benefit from treatment with EGFR-TKIs (e.g., Gefitinib and Erlotinib) for less than one year, after which there appears to be a resistance-conferring mutation in EGFR and drug resistance develops [23], especially following treatment with Gefitinib [27]. Resistance to EGFR-TKIs is inevitable due to various mechanisms (secondary mutation, activation of alternative pathways, aberrance of the downstream pathways, impairment of the EGFR-TKIs-mediated apoptosis pathway, histologic transformation, etc.) [23]. The genetic or pharmacological inhibition of AXL could prevent or overcome the acquired resistance to EGFR TKIs and restore sensitivity to erlotinib in tumor models, identifying AXL as a promising therapeutic target. Combining treatments with multiple targets may prove superior efficacy [25].

Considering the role of these three molecules in lung cancer pathology, the main goal of this study was to assess their serum concentration before and after treatment and investigate how MGP, Gas6, EGFR, and VK_1_ are modified after one cycle of chemotherapy.

## 2. Materials and Methods

All procedures were approved by the ethics committee of the University of Medicine and Pharmacy Iuliu Hațieganu Cluj-Napoca (406/11 August 2017) prior to the start of experiments. The study was conducted at the Amethyst Radiotherapy Cluj (612/6 August 2020) and the Oncology Institute Prof. Dr. Ion Chiricuță from Cluj-Napoca (185/15 July 2020). All subjects included have signed an informed consent.

### 2.1. Inclusion Criteria

We conducted an observational study in which twenty patients diagnosed with lung cancer were included. We compared in this group the serum levels of MGP, Gas6, VK_1,_ and EGFR before and after one cycle of chemotherapy. The inclusion criteria for this study were defined by patients with lung cancer, regardless of the stage of the disease, before one cycle of chemotherapy. The lung cancer diagnosis was based on thoracic computer tomography (CT) imaging, lung biopsy puncture, and pathological examination.

### 2.2. Exclusion Criteria

Age under 18, administration of vitamin K supplements treatment with anticoagulants, vitamin D antagonists, calcium, calcium antagonists, bisphosphonates, or corticosteroid drugs in the last six months were excluded from the study.

### 2.3. Sample Preparation and Determination

Blood samples obtained from freshly drawn blood were centrifuged and stored at −80 °C. An enzyme-linked immunosorbent assay (ELISA) for human VK_1_, Gas6, MGP, and EGFR was performed according to the manufacturer’s instructions (MyBioSource, Inc., San Diego, CA, USA).

Optical density was determined using a microplate reader (Stat Fax) set to 450 nm and then the concentration of human VK_1_, human Gas6, human MGP, and human EGFR were calculated.

These assays have high sensitivity and specificity for detection of VK_1_, Gas6, MGP, and EGFR. No significant cross-reactivity or interference between these parameters and analogs was observed.

The second blood sample was obtained after approximately three weeks from the first cycle of chemotherapy, and the parameters were detected using the same methods. Moreover, common blood analyses were evaluated, including complete blood count, creatinine, urea, total bilirubin, aspartate aminotransferase, alanine aminotransferase, gamma glutamyl transferase, calcium, alkaline phosphatase, lactate dehydrogenase, potassium, sodium, magnesium, and glucose.

#### CT Evaluation

Of a total of twenty patients included in this group, five had to be removed due to a lack of available pretreatment data. Using contrast-enhanced thoracic CT, we have compared the evolution of the remaining fifteen lung cancer patients before and after therapy. The interval between the initial scan and control ranged from 1 to 7 months, with an average of 3.07 and a standard deviation of 1.49 months. Patients were evaluated using RECIST 1.1 criteria, which is the gold standard for assessment of treatment response in solid tumors [28].

### 2.4. Statistical Analysis

Continuous variables were presented as mean and standard deviation (SD) or median and interquartile range, and categorical variables were presented as frequency and percentages. We performed descriptive and inferential statistical analysis to summarize the characteristics of the study population. To evaluate the proportion between nominal variables, we applied the Fisher exact test. The results of the Shapiro-Wilk normality test showed a non-Gaussian distribution; therefore, we continued to use non-parametric tests. We analyzed the strength of a linear relationship between VK_1_, Gas6, MGP, and EGFR using Spearman’s rank-order correlation test. To compare patients’ general characteristics, we employed the Mann-Whitney U test. To compare patients’ laboratory characteristics pre- and post-treatment, we employed the Wilcoxon matched pair signed rank test. The individual impacts of several confounding factors on the variance of continuous variables were assessed by building multivariate regression models. The quality of the model was described by using the accuracy of prediction and R squared. The predictors in the final regression equations were accepted according to a repeated backward-stepwise algorithm (inclusion criteria *p* < 0.05, exclusion criteria *p* > 0.10) in order to obtain the most appropriate theoretical model to fit the collected data. Data analysis was performed using SPSS v. 26.0 (Statistical Package for the Social Sciences, Chicago, IL, USA). A *p* value of < 0.05 was considered to indicate a statistically significant difference.

## 3. Results

### 3.1. General Characteristics of the Study Group

The twenty patients included in our study had a median age of 64.5 [59.75–73.5] years, and most of them were men (80%). Male patients in this study were older than the female patients, but no significant statistical difference was observed (65.5 vs. 59 years, *p* = 0.178). Four patients (20%) were non-smokers, five (25%) were former smokers, and eleven (55%) were current smokers.

There were no statistically significant differences in the gender distribution of smoker status (*p* = 0.958).

From the point of view of histopathological type of cancer, most of our study group patients presented adenocarcinoma (ten patients, 50%), followed by squamous cell carcinoma (six patients, 30%), and small cell lung cancer (four patients, 20%).

**Figure 1:**
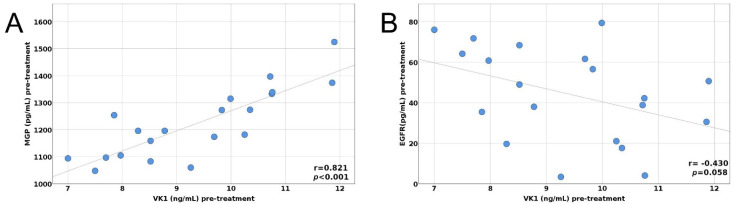
(**A**) Pre-treatment correlation between MGP and VitK_1_, (**B**) Pre-treatment correlation between EGFR and VitK_1_.

As for chemotherapy, most of the adenocarcinoma patients were treated with pemetrexed and carboplatin (five patients, 25%), and the squamous cell carcinoma patients with carboplatin (four patients, 20%) (Figure 1).

### 3.2. Biochemical and Hematological Parameters

We wanted to assess the general status of our patients before and after the chemotherapeutic treatment. The post-treatment values of white blood cells (WBC) were significantly lower compared to pre-treatment (5.2 vs. 8.13 × 10^9^/L; *p* = 0.007). We also observed a significant rise in total bilirubin and calcium levels in the post-treatment group, 0.51 vs. 0.41 mg/dL (*p* = 0.014) and 9.5 vs. 9.3 mg/dL (*p* = 0.019), respectively (Table 1).

In addition, serum levels of Gas6, EGFR, VK_1_, and MGP were compared in patients before and after chemotherapeutical treatment. The pre-treatment values of Gas6, EGFR, VK_1_, and MGP were slightly higher compared to the post-treatment values, but without reaching significant statistical differences (Table 2).

Further, pre-treatment and post-treatment correlations between MGP, Gas6, EGFR, and VK_1_ are summarized in Table 3.

**Figure 2:**
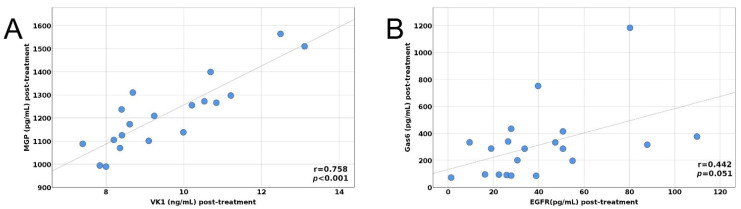
(**A**) Post-treatment correlation between MGP and VitK_1_, (**B**) Post-treatment correlation between Gas6 and EGFR.

The pre- and post-treatment correlations between MGP, Gas6, EGFR, and VK_1_ are depicted in Figure 1A,B and Figure 2A,B.

Furthermore, multivariate linear regression was employed to evaluate the independent predictor factors of Gas6 in our patients’ groups (Table 4). The predictors in the final regression equations were accepted according to a repeated backward-stepwise algorithm to obtain the most appropriate theoretical model to fit the collected data. Our results highlighted that VK_1_ is a predictor factor for Gas6 post-treatment values. Additionally, age and smoking influence the post-treatment values of Gas6. This model seems to be a good fit, explaining 89.4% of the Gas6 variance (adjusted R^2^ = 0.894).

A multivariate linear regression was performed (Table 5) to identify the independent predictor factors of MGP post-treatment values in our study group. Our analysis showed that pre-treatment values of various parameters, like MGP and VK_1,_ were predictor factors for MGP post-treatment values. Furthermore, age and smoking influence the post-treatment values of MGP. This model explains 53% of the MGP variance (adjusted R^2^ = 0.530).

Finally, we used a multivariate linear regression to identify the independent predictor factors of Gas6 post-treatment values in the study group. Our results showed that the independent factors that influenced the Gas6 values after treatment were MGP and EGFR (R^2^ = 0.405) (Table 6).

According to RECIST 1.1 criteria, one patient (6.66%) had a complete remission and one (6.66%) had an unknown complete remission with persistent imaging abnormalities. Four patients (26.67%) had a partial response, and eight (53.33%) showed minimal disease progression or amelioration. There was only one (6.66%) case of significant disease progression. According to the RECIST 1.1 criteria regarding the response to treatment, there was no significant difference in serum levels of MGP, Gas6, vitamin K_1_ and EGFR after one cycle of chemotherapy.

## 4. Discussion

Based on the main histopathological types, lung cancers are divided into two main groups: small-cell carcinoma (13 % of the cases) and non-small-cell carcinoma (83 % of the cases). Non-small-cell lung carcinoma is further subdivided into adenocarcinoma and its variants, squamous cell carcinoma, and large-cell lung carcinoma, as well as salivary gland-type tumors and sarcomatous carcinomas [1,3].

Adenocarcinoma is the most common type of non-small-cell carcinoma and the most common subtype of lung cancer in non-smokers, originating in the bronchial mucous glands [29].

In our study, adenocarcinoma represented the most frequent type of lung cancer, encountered in 50% of patients. This is correlated with the fact that most patients were non-smokers or former smokers. Despite its significant advances, chemotherapy in adenocarcinoma leaves most patients’ survival at an abysmal level [30,31,32,33,34].

Carboplatin was mostly used in our study (in total, in fifteen patients, 75%), and pemetrexed in five patients (25% of cases). Similar to our documentation from the scientific literature [35,36,37], the combination most commonly used was pemetrexed and carboplatin (five patients, 25%).

We observed a significant rise in total bilirubin, illustrating the impairment of hepatic function after the chemotherapy. During cancer chemotherapy, the drug-related toxicity can be unpredictable and alter the hepatic function and the drug clearance. A bilirubin level greater than 5.0 mg/dl is generally regarded as an absolute contraindication to administering cytotoxic chemotherapy [38].

In addition, in our study, we looked at serum levels of common laboratory parameters before and after treatment. We noticed a significant rise in calcium levels in the post-treatment group, which can illustrate the possible presence of bone metastases. Bone is a common site of metastatic cancer spread, mostly in adenocarcinoma, and can lead to pathological fractures, nerve root compression at the level of spinal cord vertebrae, or hypercalcemia of malignancy [39].

Cellular differentiation in cancer and tumor progression [40] has recently been linked to MGP expression. In addition, the same study proved that MGP might contribute to increased cancer resistance to chemotherapeutic drugs by augmenting the interaction of stromal cancerous cells with the extracellular matrix components [40]. It has also been shown that an increased expression of MGP is linked to the respective chemotherapeutic drugs [40,41].

In our study, MGP serum values were strongly correlated with VK_1_ values, only before therapy. Moreover, MGP decreased insignificantly after chemotherapy in our study. Since MGP is a marker for extrahepatic vitamin K insufficiency, we found that after the first chemotherapy cycle, patients had no deficiency in vitamin K.

It was observed that Gas6 is frequently overexpressed in lung cancer cells and is found in the plasma [42,43], and Gas6 is associated with cell growth of stromal cancerous cells and tumor progression [44]. Therapeutic agents targeting Gas6 and AXL have been developed, and promising results have been observed in both preclinical and clinical settings when such agents are used alone or in combination [45].

In contrast to Kanzaki et al.’s [19] murine models, where plasma levels of Gas6 and its expression in stromal cancerous cells increased after cisplatin chemotherapy, we found an insignificant decrease in serum level of Gas6 after the first chemotherapy cycle with cisplatin in our study Moreover, compared to the study of Nonagase et al. [46], the plasma Gas6 concentration after EGFR–tyrosine kinase inhibitors (TKIs) was increased. These contradictory results could be explained by the alterations induced by chemotherapy, which could influence the behavior of cancer cells. In the tumor stromal microenvironment, cancer-associated fibroblasts play an important role, and Gas6 could promote non-small cell lung cancer. In a murine model, Kanzaki et al. [19] found that Gas6 expression by cancer-associated fibroblasts was upregulated following cisplatin treatment. Moreover, Gas6 expression might be influenced by intratumoral hypoperfusion during chemotherapy. On the other hand, Gas6 expression is increased after serum starvation in a human lung CAF line. In clinical samples, stromal Gas6 expression increased after chemotherapy. Plasma samples were obtained from EGFR-mutated NSCLC patients before or after treatment with first or second-generation EGFR-TKIs in the study by Nanogase et al. [46].

In the current literature, EGFR gene mutations and protein overexpression are involved in the development and progression of lung cancer [23,47]. However, EGFR’s value as a prognostic biomarker for survival outcomes is not yet established. There are no consistent results regarding the predictive value of EGFR serum levels in lung cancer patients and, more importantly, its ability to predict therapeutic response to anti-EGFR drugs [48,49,50]. Mohan et al. [48] published a study in 2020 on the measurement of serum levels of EGFR mRNA expression, attempting to emphasize its value as a predictor of response to treatment and survival in non-small cell lung cancer types. In that study, most of the patients had advanced lung cancer (70.6%), and serum EGFR mRNA expression was considered a marker for predicting treatment response and survival outcomes in non-small-cell carcinoma [48]. Another study, which used an ELISA assay to measure and compare EGFR serum protein and circulating mRNA levels, found that both the protein serum level and gene expression of EGFR were higher in patients with lung cancer [51]. Our study found a moderately negative correlation between VK1 and EGFR pre-treatment values, the relationship between them being marginally significant.

To identify possible correlations between the post-treatment values of the parameters investigated, in our multivariate linear regression for Gas6 post-treatment value, we obtained a stronger correlation between the post-treatment values of Gas6 and EGFR. The increase of one unit of measurement of EGFR leads to an increase of the Gas6 value by 4.245 units (Table 6).

The study aimed not to highlight the variations compared to the general population, but to highlight the modification of the four parameters (Gas6, MGP, EGFR, and vitamin K) pre- and post-treatment and to underline the possible mechanisms associated with the treatment.

Certain limitations of the present study should be considered. First, the sample size was relatively small, and the study design was observational. Another potential limitation was that serum MGP, Gas6, and EGFR levels were assessed only after the first cycle of chemotherapy. Further studies are required for the evaluation of these parameters after more than one cycle of chemotherapy. Moreover, different stages of the disease may influence the serum concentration of MGP, Gas6, and EGFR after the first dose of chemotherapy. Maybe these variations in tumor characteristics could be responsible for the fluctuation of biomarker values and their consequent non-significance.

However, the data obtained represents preliminary results that can offer new opportunities. According to the RECIST 1.1 criteria, there was no statistically significant difference in serum levels of MGP, Gas6, and EGFR after one cycle of chemotherapy. Our study was the first that evaluated the serum levels of those three proteins in lung cancer patients after one cycle of chemotherapy.

## 5. Conclusions

Lung cancer is a complex and heterogeneous disease that needs a multidisciplinary approach for diagnosis, classification, and therapy. The serum markers profiling cancer and targeted therapy represent the future main directions for efficient cancer therapy.

Between VKDPs, the pre-treatment values of MGP correlate strongly with the vitamin K_1_ level.

The post-treatment value of EGFR is a stronger independent factor that correlates positively with the Gas6 post-treatment values.

Our pilot study did not find any serum modification of these proteins in the serum samples after one dose of chemotherapy. Based on RECIST 1.1 criteria, these proteins (MGP, Gas6, and EGFR) could not be markers for response to treatment in patients treated with cisplatin.

## Figures and Tables

**Table 1:** General laboratory characteristics pre- and post-treatment.

Variable	Pre-Treatment	Post-Treatment	*p* ^a^
Hemoglobin (g/dL)	12.25 [11.82–14.35]	12.4 [10.87–14.15]	0.695
Platelet count (×10^9^/L)	249.5 [183.5–312]	234 [189.7–331]	0.586
WBC (×10^9^/L)	8.13 [5.28–10.28]	5.2 [4.09–7.38]	0.007 *
Creatinine (mg/dL)	0.92 [0.7–1.07]	0.86 [0.73–1.11]	0.763
Urea (mg/dL)	36.2 [29.2–49.7]	36.2 [26.4–41.5]	0.163
Total Bilirubin (mg/dL)	0.41 [0.29–0.54]	0.51 [0.32–0.61]	0.014 *
ASAT (U/L)	21 [16.2–24.9]	18 [13–23.5]	0.669
ALAT (U/L)	25.5 [11.5–36.2]	22 [17.5–27]	0.722
GGT (U/L)	47 [26.2–116.5]	60.5 [26.5–81.5]	0.311
Calcium (mg/dL)	9.3 [8.9–9.8]	9.5 [9.1–10]	0.019 *
Neutrophils (%)	69.2 [58.65–81.03]	58.25 [50.38–72.95]	0.032 *
Lymphocytes (%)	18.4 [11.07–29.8]	27.75 [12.72–34.9]	0.09
ALP (U/L)	92 [81–125.5]	98 [79.5–125.7]	0.401
LDH (U/L)	211.5 [182.2–280.2]	166 [146.2–253.5]	0.094
K (mmol/L)	4.5 [4–4.9]	4.5 [3.6–4.8]	0.220
Na (mmol/L)	138 [136–140]	138 [135–138]	0.090
Mg (mg/dL)	1.8 [1.7–1.9]	1.8 [1.7–2]	0.638
Glucose (mg/dL)	101 [95–115]	102 [95–123]	0.816

**Legend:** WBC—White blood cell, ASAT—Aspartate aminotransferase; ALAT –Alanine aminotransferase; GGT—Gamma glutamyl transferase; ALP—Alkaline phosphatase; LDH—Lactate dehydrogenase; K—Potassium; Na—Sodium; Mg—Magnesium; ^a^ Wilcoxon matched-pair signed rank test; * significance threshold reached.

**Table 2:** Specific laboratory characteristics pre- and post-treatment.

Variable	Pre-Treatment	Post-Treatment	*p* ^a^
Gas6 (pg/mL)	311.5 [121.1–368.5]	286.7 [94.5–366.8]	0.647
EGFR (pg/mL)	45.6 [23.4–63.5]	32.1 [23.3–50.7]	0.401
VK_1_ (ng/mL)	9.4 [8–10.6]	8.89 [8.4–10.6]	0.614
MGP (pg/mL)	1196 [1099–1328.5]	1191 [1102–1290.7]	0.709

**Legend:**^a^ Wilcoxon matched-pair signed rank test; Gas6—Growth arrest specific 6; EGFR—Epidermal growth factor receptor; VK_1_—VitaminK_1_; MGP—Matrix Gla protein.

**Table 3:** MGP pre- and post-treatment correlations.

Pre- and Post-Treatment Variables	MGP Pre-Treatment		MGP Post-Treatment	
	r (95%CI)	*p* ^a^	r (95%CI)	*p* ^a^
Gas6 (pg/mL)	−0.167 (−0.507; −0.223)	0.482	0.018 (−0.417; 0.498)	0.940
EGFR (pg/mL)	−0.316 (−0.784; 0.146)	0.175	0.087 (−0.377; 0.533)	0.717
VK_1_ (ng/mL)	0.821 (0.523; 0.954)	<0.001 *	0.857 (0.609; 0.950)	*p* < 0.001 *

**Abbreviations:** Gas6—Growth arrest specific 6; EGFR—Epidermal growth factor receptor; VK_1_—VitaminK_1_; MGP—Matrix Gla protein; ^a^ Spearman’s rank-order correlation test; * significance threshold reached.

**Table 4:** Multivariate linear regression for Gas6 post-treatment values.

Variable	β	Standard Error	*p*	95%CI for β
Gas6 (pg/mL) pre-treatment	0.853	0.128	<0.001	0.581; 1.124
Age	9.587	3.395	0.018	2.023; 17.151
VK_1_ (ng/mL) pre-treatment	−79.712	22.650	0.006	−130.179; −29.245
Smoking	66.233	33.419	0.076	8.229; 140.694

**Abbreviations:** Gas6—Growth arrest specific 6; VK_1_—VitaminK_1._

**Table 5:** Multivariate linear regression for MGP post-treatment values.

Variable	β	Standard error	*p*	95%CI for β
MGP (pg/mL) pre-treatment	−1.299	0.391	0.005	−2.132; −0.466
EGFR (pg/mL) pre-treatment	5.070	2.180	0.034	0.424; 9.717
VK_1_ (ng/mL) pre-treatment	130.985	37.702	0.003	50.624; 211.345

**Abbreviations:** Gas6—Growth arrest specific 6; EGFR—Epidermal growth factor receptor; VK_1_—VitaminK_1._

**Table 6:** Post-treatment values for Gas 6 after multivariate linear regression.

Variable	β	Standard Error	*p*	95%CI for β
MGP (pg/mL) post-treatment	−0.448	0.169	0.017	−0.806; −0.090
EGFR (pg/mL) post-treatment	4.245	1.740	0.027	0.556; 7.935

**Abbreviations:** MGP—Matrix Gla protein; EGFR—Epidermal growth factor receptor; VK_1_—Vitamin K_1._

## Data Availability

Not applicable.
